# CBCT-based synthetic CT generated using CycleGAN with HU correction for adaptive radiotherapy of nasopharyngeal carcinoma

**DOI:** 10.1038/s41598-023-33472-w

**Published:** 2023-04-24

**Authors:** Chen Jihong, Quan Kerun, Chen Kaiqiang, Zhang Xiuchun, Zhou Yimin, Bai penggang

**Affiliations:** 1 Department of Radiation Oncology, Clinical Oncology School of Fujian Medical University, Fujian Cancer Hospital, Fuzhou, 350014 Fujian China; 2 Department of Radiation Oncology, Xiangtan City Central Hospital, Xiangtan, 411100 Hunan China; 3 School of Nuclear Science and Technology, University of South China, Hengyang, 421001 China

**Keywords:** Biophysics, Head and neck cancer, Radiotherapy

## Abstract

This study aims to utilize a hybrid approach of phantom correction and deep learning for synthesized CT (sCT) images generation based on cone-beam CT (CBCT) images for nasopharyngeal carcinoma (NPC). 52 CBCT/CT paired images of NPC patients were used for model training (41), validation (11). Hounsfield Units (HU) of the CBCT images was calibrated by a commercially available CIRS phantom. Then the original CBCT and the corrected CBCT (CBCT_cor) were trained separately with the same cycle generative adversarial network (CycleGAN) to generate SCT1 and SCT2. The mean error and mean absolute error (MAE) were used to quantify the image quality. For validations, the contours and treatment plans in CT images were transferred to original CBCT, CBCT_cor, SCT1 and SCT2 for dosimetric comparison. Dose distribution, dosimetric parameters and 3D gamma passing rate were analyzed. Compared with rigidly registered CT (RCT), the MAE of CBCT, CBCT_cor, SCT1 and SCT2 were 346.11 ± 13.58 HU, 145.95 ± 17.64 HU, 105.62 ± 16.08 HU and 83.51 ± 7.71 HU, respectively. Moreover, the average dosimetric parameter differences for the CBCT_cor, SCT1 and SCT2 were 2.7% ± 1.4%, 1.2% ± 1.0% and 0.6% ± 0.6%, respectively. Using the dose distribution of RCT images as reference, the 3D gamma passing rate of the hybrid method was significantly better than the other methods. The effectiveness of CBCT-based sCT generated using CycleGAN with HU correction for adaptive radiotherapy of nasopharyngeal carcinoma was confirmed. The image quality and dose accuracy of SCT2 were outperform the simple CycleGAN method. This finding has great significance for the clinical application of adaptive radiotherapy for NPC.

## Introduction

Nasopharyngeal carcinoma (NPC) is one of the most common malignancies in Southeast Asia and China^1^. In modern intensity-modulated radiation therapy (IMRT) and volumetric modulated arc therapy (VMAT), adequately delivering dose to the target volumes while sparing the critical organs at risk (OAR) is key to the success of radiation therapy for NPC^2,3^. Adaptive radiation therapy (ART) may be crucial for radiotherapy of NPC^4^. Accurate acquisition of 3D volume images to monitor patient-specific variation in the radiotherapy process is essential in ART. Repeated scans with fan beam based CT are potentially most accurate image acquisition for ART. However, it will increase radiation exposure, delay or prolong treatment course, add logistic burdens to the patients and also increase the workload of the clinic. Recently, Cone-beam CT (CBCT) image acquisition with an on board imaging system equipped on the treatment delivery has been widely used in patient setup and monitoring of anatomical changes during the treatment^5^. However, the inferior image quality compared to conventional CT images and the uncalibrated Hounsfield Units (HU) of CBCT have been limiting its usage in ART due to the poor target and OAR delineation and incorrect dose calculation^6^.

Improving CBCT imaging quality and HU fidelity has been extensively studied^7–10^. A primitive method is to calibrate the electron density (ED) value of the CBCT images using the HU-ED curve obtained from a commercial phantom^7^. More robust methods, such as histogram-matching based solutions^11^, voxel-to-voxel mapping using deformable image registration (DIR)^8^ and Monte-Carlo (MC) based methods^9^, have been proposed for CBCT correction. It was reported that the dosimetric accuracy of CBCT corrected by an automated patient-specific calibration method was comparable to recalculation on conventional CT data sets for head-and-neck patients^10^.

More recently, deep learning (DL) methods such as U-net CNN^12–14^ and GAN^15,16^ have already been implemented widely in the generation of synthetic CT (sCT). In particular, Cycle-consistent adversarial network (CycleGAN) is one of the most commonly used methods for CBCT to CT transformation, as it does not require paired information of the training data^17–21^. Liang et al. developed a CycleGAN network to synthesize CT images from CBCT images for head-and-neck cancer patients, and the sCT images were both visually and quantitatively similar to real CT images^17^. Kida et al.^18^ indicated that CycleGAN could produce high quality CT-mimicking images from CBCT images while preserving anatomical structures for prostate cancer patients. Sun et al.^19^ proved that 3D CycleGAN improved electronic density and anatomical structure delineation accuracy, from 2D CycleGAN. DL generated sCT images have been reported useful in dose calculations in ART applications^22–24^. Their accuracy in clinical dose calculations for NPC^16^ and prostate cancer radiation therapy^22^ were preliminarily verified.

In this study, we report a hybrid use of both tradiation and DL methods in the correction of CBCT images for the application in ART. HU of the CBCT images for NPC patients were firstly corrected by a commercial phantom. SCT images were then generated by a CycleGAN from the original CBCT images, and their resulting image quality and dosimetric accuracy of the two sets of sCT images were evaluated.

## Material and methods

### Image acquisition and processing

52 NPC patients receiving radiotherapy in Fujian Cancer Hospital from 2020 to 2021 were included in this study. This study has been approved by the ethics committee of Fujian Cancer Hospital (ethics number: SQ2020-043-01) and all patients provided written informed consent prior to enrollment in the study. All methods were performed in accordance with the Declaration of Helsinki as well as relevant guidelines and regulations. During simulation for treatment planning, CT images were obtained on a Brilliance CT Big Bore (Philips Medical Systems Inc., Cleveland, OH, USA), with a head neck protocol (120 kVp, 225 mA). The CT image slice had a dimension of 512 × 512 pixels, with a voxel resolution of 1.14 × 1.14 × 3 mm^3^. All CBCT images were acquired before the patients’ first radiotherapy on XVI of an Elekta Axesse accelerator, with a tube voltage of 120 kV and an exposure current of 25 mA. The dimension of CBCT image slice was 410 × 410 pixels with resolution of 1 × 1 × 1 mm^3^.

CT images and CBCT images were rigidly registered with a benchmark of CBCT images, using an open source-software 3D-Slicer^25^. Then the axial aligned CT images were resampled to CBCT images voxel and size, called RCT as a reference standard for image evaluation. Binary masks were created based on threshold segmentation and morphological processing methods to avoid the adverse impact from non-anatomical structures during the process of training. The voxel values of images were clipped to the range of [− 1000, 2000], while the voxel values of regions outside the masks were set to − 1000 HU.

Before the training of CycleGAN model, each RCT and CBCT images were cropped from the image center to the size of 256 × 256 and the CT value were normalized to [− 1, 1]. 41 patients were randomly chosen for the training set and the remaining 11 patients were used in validation. 264 slices were taken from each patient’s dataset. Therefore the training and validation dataset consisted of 10,824 and 2904 CT and CBCT slices, respectively. Due to GPU memory limitations, a two-dimensional CycleGAN model is adopted in this study.

### Calibration of HU by phantom

The CIRS model 062 (CIRS Tissue Simulation Technology, Norfolk, VA, USA) was scanned with the same Big Bore CT and the same CBCT on the linear accelerator, with the same acquisition parameters. For each scan, the average HU number of each material insertion (electron density relative to water of 1.00, 0.20, 0.50, 0.97, 0.99, 1.06, 1.07, 1.16 and 1.61) was read out in the central slice of the phantom. Then the average HU number in the CT scan and CBCT scan was plotted against the known electron density, respectively. HU of the CBCT images were corrected based on these two curves, by an in-house program to make the corrected CBCT images (CBCT_cor).

### CycleGAN method

**Figure 1:**
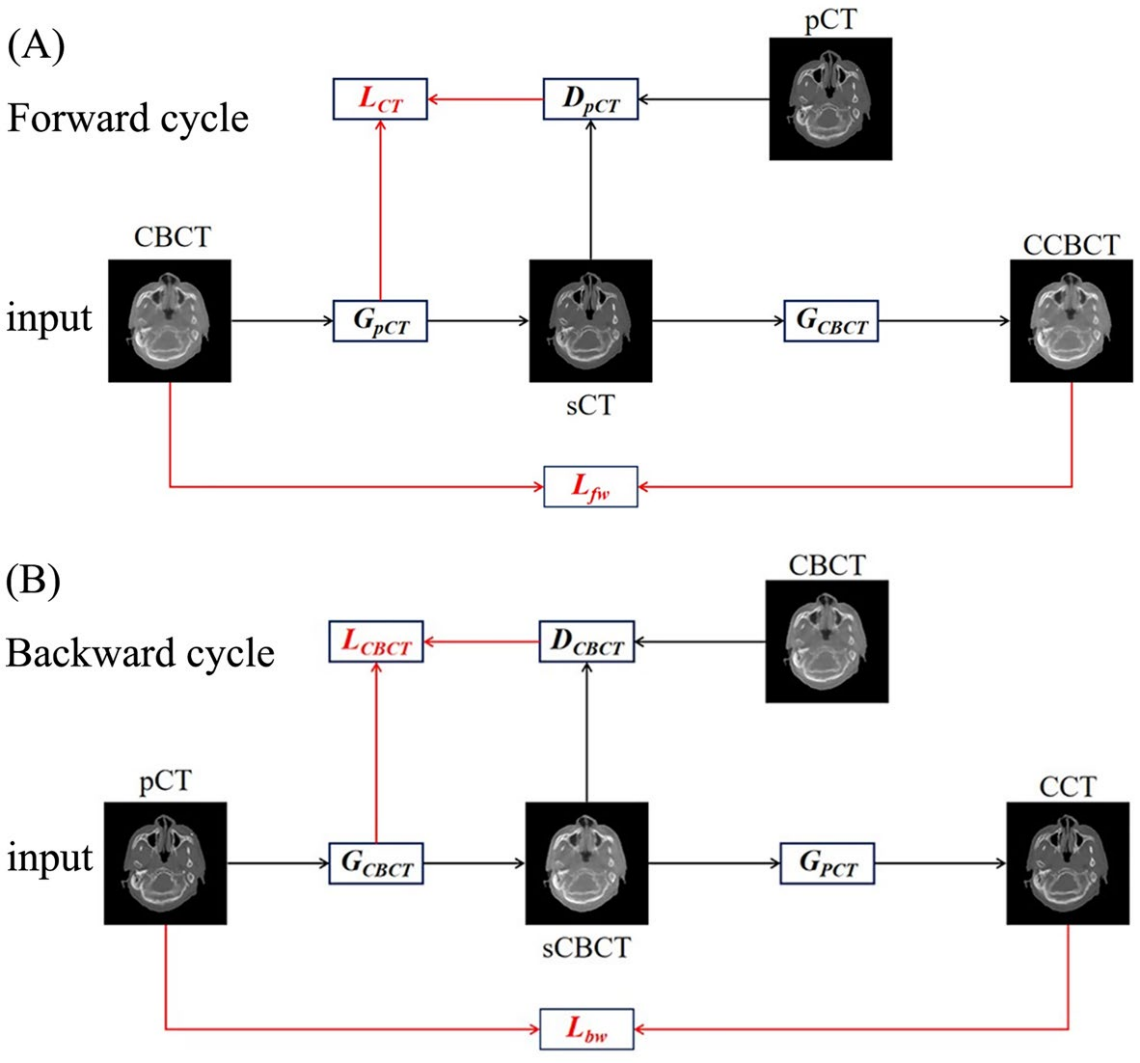
Illustration of cycle-consistent generative adversarial network (CycleGAN).

As shown in Fig. 1, the CycleGAN model includes two generators and two discriminators. In the forward cycle, Generator-RCT (G_RCT_) generates sCT from CBCT, and then Generator-CBCT (G_CBCT_) generates Cycle CBCT (CCBCT) from sCT. While in the backward cycle, G_CBCT_ generates synthesized CBCT (sCBCT) from RCT, and then G_RCT_ generates Cycle CT (CCT) from sCBCT. The two discriminators, D_RCT_ and D_CBCT_, were used to determine whether sCT and sCBCT were real images. Loss function of CycleGAN was consisted of adversarial loss and cycle consistency loss. The adversarial losses for the two cycles are

1 $$ L_{CT} = E_{RCT} \left[ {\left( {1 - D_{RCT} (RCT)} \right)^{2} } \right] + E_{CBCT} \left[ {\left( {D_{RCT} \left( {G_{RCT} (CBCT)} \right)} \right)^{2} } \right] $$

and

2 $$ L_{CBCT} = E_{CBCT} \left[ {\left( {1 - D_{CBCT} (CBCT)} \right)^{2} } \right] + E_{RCT} \left[ {\left( {D_{CBCT} \left( {G_{CBCT} (RCT)} \right)} \right)^{2} } \right] $$

The cycle consistency losses for the two cycles are

3 $$ L_{fw} = E_{CBCT} \left[ {\left\| {CBCT - G_{CBCT} (G_{RCT} (CBCT))} \right\|_{1} } \right] $$

and

4 $$ L_{bw} = E_{RCT} \left[ {\left\| {RCT - G_{RCT} \left( {G_{CBCT} (RCT)} \right)} \right\|_{1} } \right] $$

Thus, combining these two kinds of losses, the full objective is:

5 $$ L_{cyclegan} = L_{CT} + L_{CBCT} + \lambda \left( {L_{fw} + L_{bw} } \right) $$

### Network structure and parameters

The generator contains an encoding layer, a conversion layer and a decoding layer. The encoder reduces the number of spatial dimensions and identifies the features of the input image. The conversion layer, which consists of nine layers of ResNet module^26^, will then change to its eigenvectors. The decoder repairs the spatial dimensions of the object and generates a synthesized image. The discriminator is a binary network with outputs between [0, 1]. The mode is trained with Adam optimizer^27^ from Tensorflow^28^. The learning rate decays linearly after 20 epochs with an initial value of 0.0002, while the momentum term β1 and β2 are set to 0.5. The other parameters are set as follows: λ = 10, batch-size = 2, epoch = 100. The original CBCT images and CBCT_cor images are used to train the model respectively. In the following text, SCT1 is generated by the CycleGAN model from original CBCT images while SCT2 is generated from the CBCT_cor.

### Evaluation

In this study, the patients in the validation set were used to evaluate the improvement of image quality. The Mean Absolute Error (MAE) and Mean Error (ME) for CBCT, CBCT_cor, SCT1 and SCT2 versus RCT were calculated in the region of Binary masks, respectively. Meanwhile, HU profiles were also compared for these types of images while a side-by-side comparison was performed.

Volumetric modulated arc therapy (VMAT) plans of the patients in the validation set were generated on the RCT images. The prescribed dose were 69.96 Gy, 60.06 Gy and 56.1 Gy to the planning target volumes of primary nasopharyngeal tumor and definitive bilateral lymph nodes (PTV6996), high risk region (PTV6006), low risk region and bilateral low-risk nodal regions (PTV5610) in 33 fractions, respectively. The contours were copied from the RCT images to the CBCT, CBCT_cor, SCT1 and SCT2 images via rigid registration. Dose calculation was performed with the Pinnacle^3^ (version 16.2, Philips Radiation Oncology Systems, Madison, WI).

The comparison of dose distribution was performed among CBCT_cor, RCT, SCT1 and SCT2 images. Several dosimetric parameters were collected for quantitative comparisons. For PTVs, the D_2_ (the dose corresponding to 2% of volume), D_mean_ (the mean dose) and D_98_ (the dose corresponding to 98% of volume) were recorded. For OARs, D_mean_ or D_max_ (the max dose) were compared. The global 3D gamma passing rates were also calculated by the radiotherapy module of 3D-Slicer with criteria of 3%/3 mm and 2%/2 mm, with 10% dose threshold, respectively.

The Wilcoxon’s signed rank test was carried out (between SCT2 and CBCT, SCT2 and CBCT_cor, SCT2 and SCT1,) for MAE, ME, gamma pass rate and dosimetric parameters previously described. Statistical Package for the Social Sciences (SPSS 21.0; SPSS Inc., Chicago, IL, USA) was used to perform these tests and *P* < 0.05 was considered statistically significant.

## Results

### Side-by-side comparison

**Figure 2:**
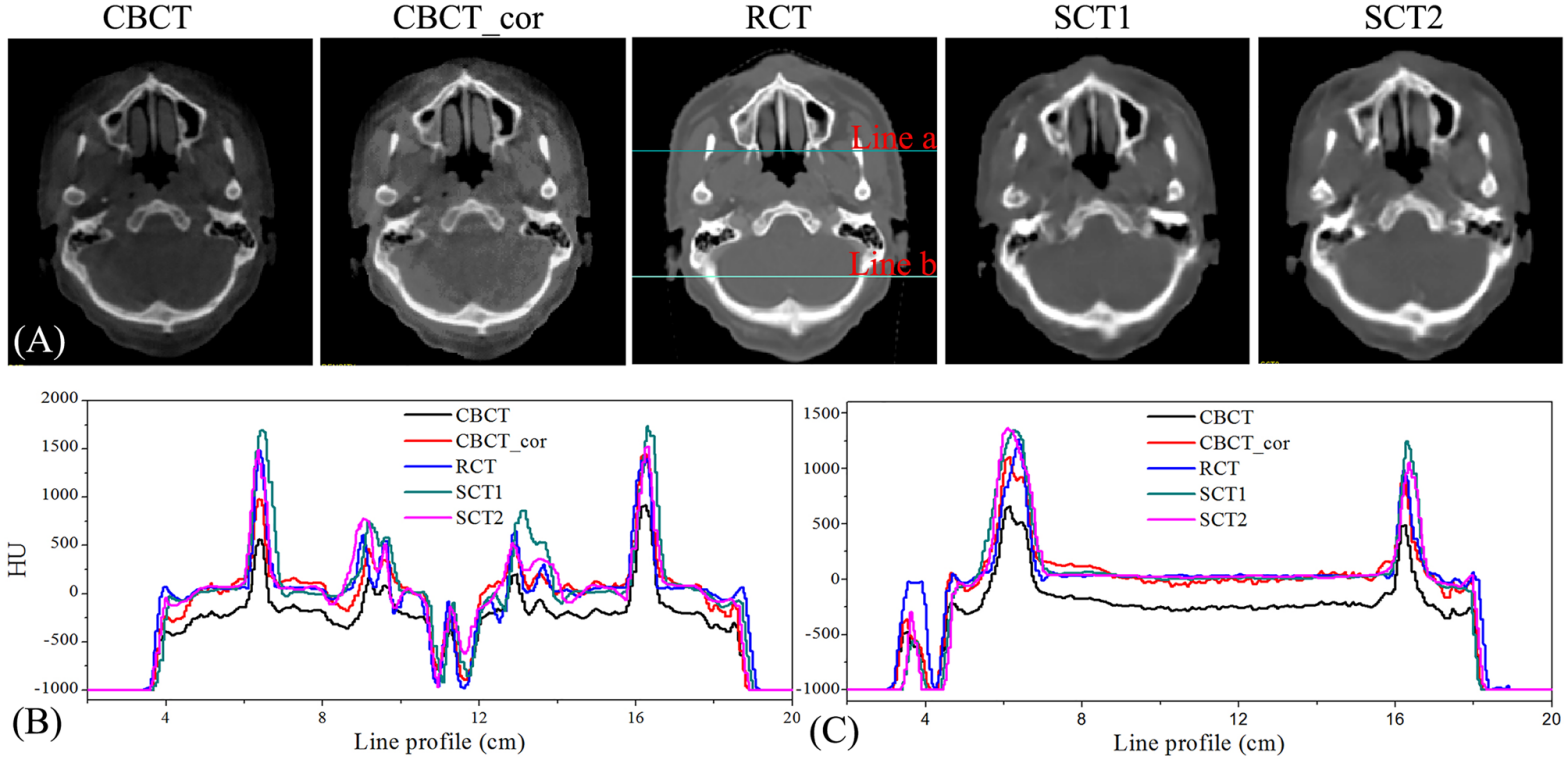
(**A**) The side-by-side comparison of CBCT, CBCT_cor, RCT, SCT1 and SCT2 for a validation patient; (**B**) the line profile of line a; (**C**) the line profile of line b.

Figure 2 shows CBCT, CBCT_cor, RCT, SCT1 and SCT2 images from one patient of validation set. The image quality of SCT1 and SCT2 were significantly better than that of the CBCT and CBCT_cor. As shown in Fig. 2A, both of SCT1 and SCT2 images generated by CycleGAN model could remove the scattering artifacts of CBCT images. Meanwhile, the line profiles of different areas for this patient were plotted. In the profile of line A which passes though soft tissue, bone and cavity areas, sCT images especially for SCT2 HU values were well corrected to the HU values of RCT. In the profile of line B which passes though brain tissue area, the SCT1 and SCT2 HU values were smooth and well corrected to the RCT HU values too. Nevertheless, the CBCT and CBCT_cor HU values were obvious noisy.

### The MAE and ME evaluation

**Figure 3:**
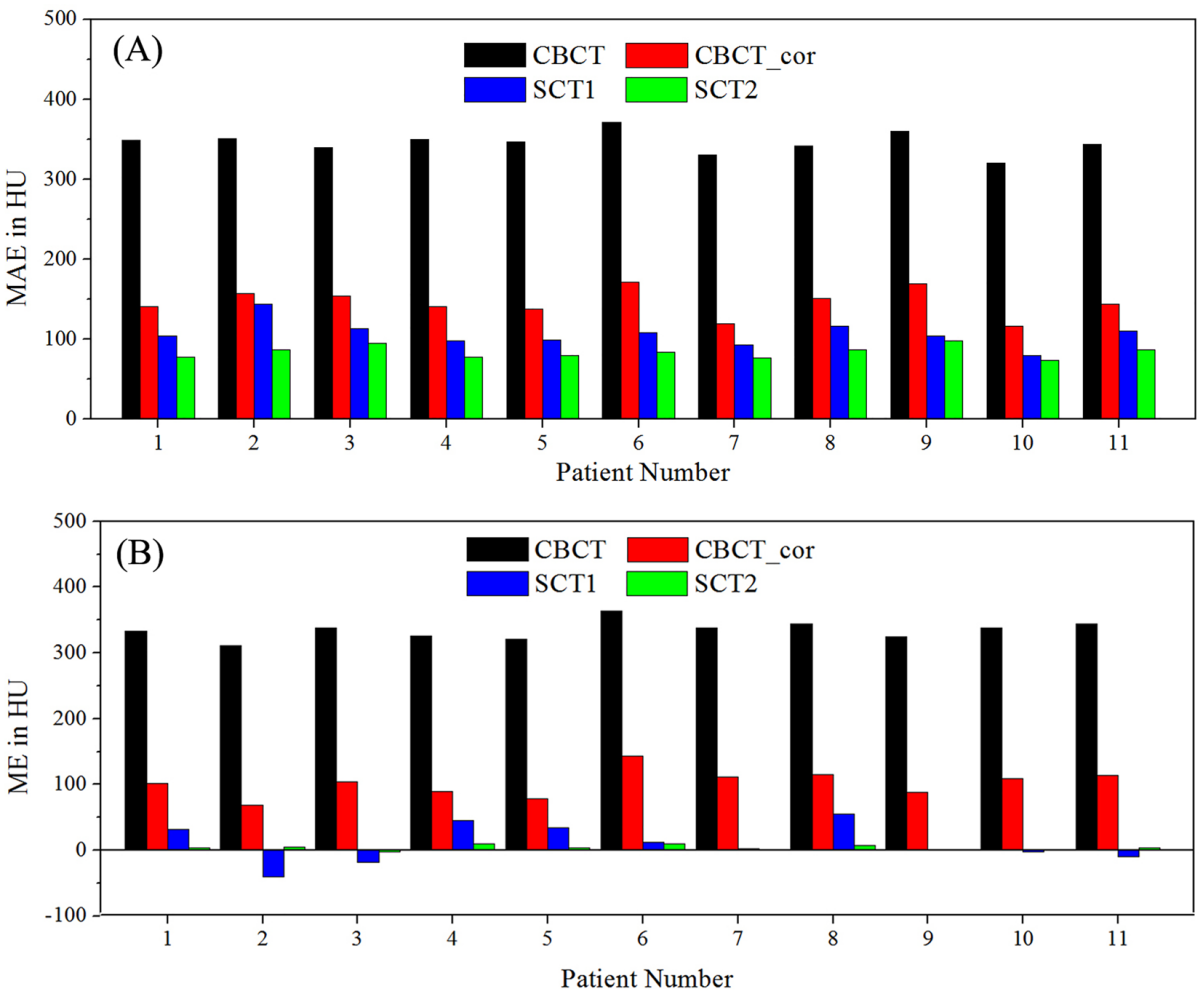
MAE and ME comparisons for each patient in validation cases. (**A**) MAE, (**B**) ME.

The results of MAE and ME comparisons between the CBCT, CBCT_cor, SCT1 and SCT2 images against RCT images for all 11 validation cases are listed in Table 1 and Fig. 3. For both MAE and ME, the result of SCT2 images was the smallest, follow by SCT1. In addition, as shown in Fig. 3, the MAE for each patient between SCT2 and RCT is less than that between SCT1 and RCT. The range of MAE improved from (79, 143) to (74, 97), which suggested that corrected CBCT images can help improve training results.

**Table 1:** MAE and ME results for four kinds of images against RCT images from all validation cases.

	MAE (HU)	*P* value	ME (HU)	*P* value
CBCT	346.11 ± 13.58	0.003	334.80 ± 13.88	0.003
CBCT_cor	145.95 ± 17.64	0.003	101.92 ± 20.39	0.003
SCT1	105.62 ± 16.08	0.004	10.07 ± 28.95	0.424
SCT2	83.51 ± 7.71	–	3.65 ± 4.20	–

*MAE* mean absolute error, *ME* mean error.

### Dose distribution comparison

**Figure 4:**
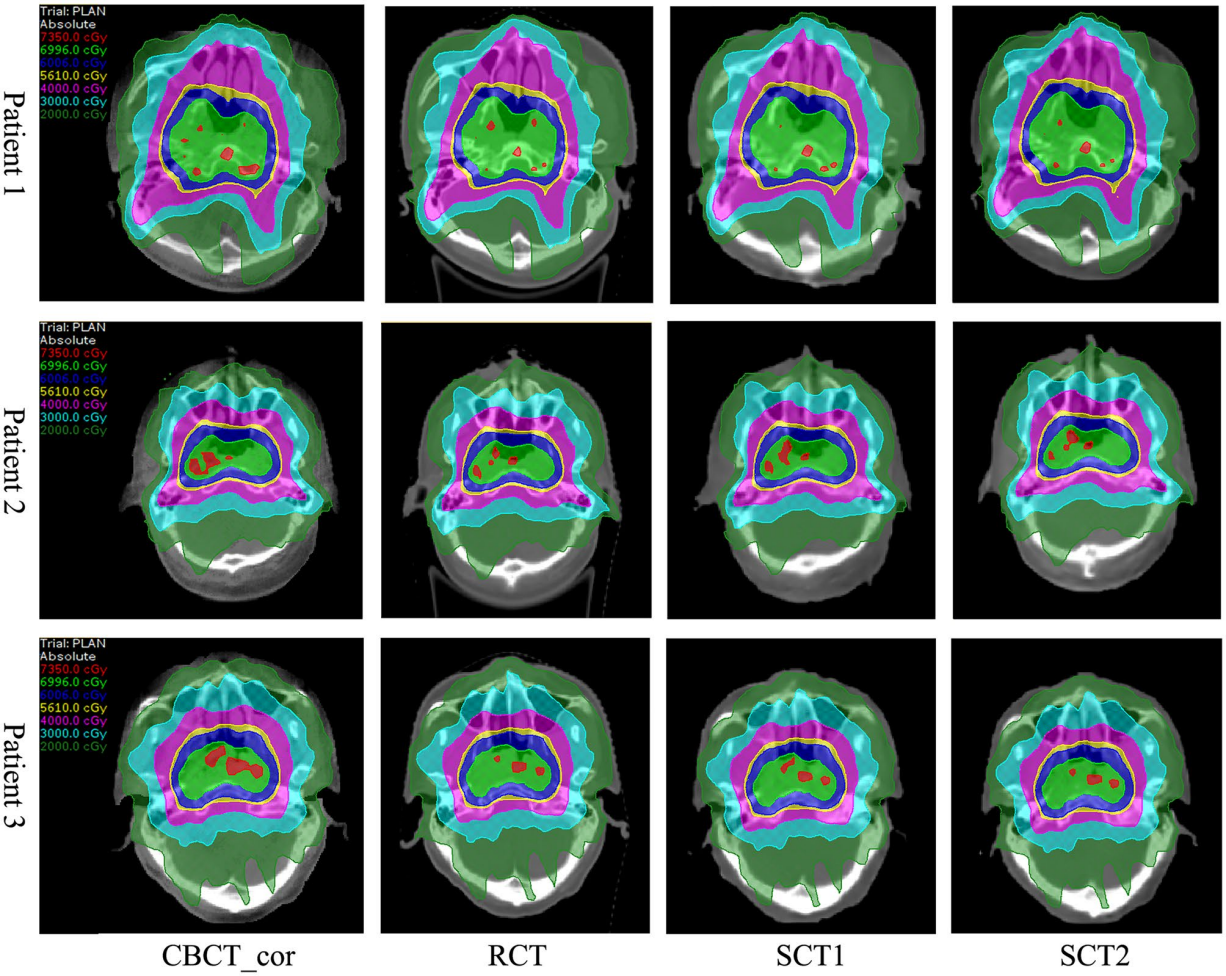
The dose distributions for three validation patients on CBCT_cor, RCT, SCT1 and SCT2 were displayed.

Figure 4 shows the dose distribution based on CBCT_cor, RCT, SCT1 and SCT2 images for three validation patients. The distribution of isodose lines on SCT2 was closest to that on RCT, followed by SCT1. Moreover, the isodose lines of CBCT_cor such as 7350 cGy were significantly different from that of RCT.

The average relative dosimetric difference for the CBCT, CBCT_cor, SCT1 and SCT2 compared to RCT of all validation patients were listed in Table 2. The average dosimetric difference (6.5 ± 8.7%) was considerable when calculated from the uncorrected CBCT images. For most targets and OARs, the relative dosimetric differences for SCT2 were least compared to the RCT (0.6% ± 0.6%). Respectively, the average differences for CBCT_cor and SCT1 were (2.7% ± 1.4%) and (1.2% ± 1.0%) respectively.

**Table 2:** The average relative dosimetric difference for the CBCT, CBCT_cor, SCT1 and SCT2 compared to RCT of all validation patients (mean ± SD).

OARs	Index	CBCT	CBCT-cor	SCT1	SCT2
PTV6996	D_98_ (%)	4.6 ± 2.0	1.9 ± 0.6*	1.0 ± 0.2	1.0 ± 0.4
	D_mean_ (%)	11.5 ± 2.1*	4.3 ± 0.3*	0.6 ± 0.3	0.5 ± 0.4
	D_2_ (%)	7.3 ± 2.3*	2.7 ± 0.4*	1.7 ± 0.3	1.7 ± 0.4
PTV6006	D_98_ (%)	19.5 ± 2.3*	3.6 ± 0.3	1.0 ± 0.4	0.9 ± 0.4
	D_mean_ (%)	7.2 ± 2.0*	3.8 ± 0.6*	1.5 ± 0.2	1.4 ± 0.4
	D_2_ (%)	8.0 ± 2.4*	3.1 ± 0.4	2.1 ± 0.3	2.0 ± 0.4
PTV5610	D_98_ (%)	18.3 ± 2.7*	2.3 ± 0.4	1.6 ± 0.4*	0.7 ± 0.5
	D_mean_ (%)	3.3 ± 2.1*	1.4 ± 0.6*	0.8 ± 0.1	0.7 ± 0.4
	D_2_ (%)	7.2 ± 2.4*	2.6 ± 0.4*	1.5 ± 0.3	1.5 ± 0.4
Parotid	D_mean_ (%)	6.4 ± 4.7*	1.0 ± 1.4*	1.2 ± 0.3*	0.7 ± 0.9
Brain stem	D_max_ (%)	7.9 ± 3.0*	2.9 ± 0.5*	1.9 ± 0.4	1.6 ± 0.6
Spinal cord	D_max_ (%)	7.6 ± 4.5*	2.3 ± 0.7*	1.6 ± 0.6	1.5 ± 0.8
Lens	D_max_ (%)	11.3 ± 3.7*	4.8 ± 6.0*	2.3 ± 4.7	1.9 ± 0.7
Optic chiasm	D_max_ (%)	7.1 ± 3.8*	2.1 ± 0.6	1.9 ± 0.5	1.6 ± 0.7
Optic nerve	D_max_ (%)	5.2 ± 4.5*	2.0 ± 0.7*	1.5 ± 0.6	1.3 ± 0.8
Thyroid	D_mean_ (%)	6.5 ± 8.7	1.9 ± 2.7	0.6 ± 0.6*	1.3 ± 1.7
Average of dose metric (%)		6.5 ± 8.7	2.7 ± 1.4	1.2 ± 1.0	0.6 ± 0.6

PTV6996, PTV6006 and PTV5610 represent the planning targets with prescription of 69.96 Gy, 60.06 Gy and 56.1 Gy, respectively. D_98_: the dose corresponding to 98% of volume. D_mean_: the mean dose. D_2_: the dose corresponding to 2% of volume. D_max_: the max dose. Significant differences (*p* ≤ 0.05) are displayed using the symbol*.

### 3D gamma analysis

The result of 3D gamma analysis was shown in Table 3. Regardless of the 3 mm/3% or 2 mm/2% criteria, the gamma passing rate of SCT2 compared with RCT (98.7% and 97.1%, respectively) was marginally higher than that of SCT1 (97.7% and 95.7%, respectively). Moreover, both of STC1 and SCT2 have significantly higher passing rates than that of CBCT_cor.

**Table 3:** 3D gamma analysis of the CBCT, CBCT_cor, SCT1 and SCT2 compared with RCT (mean ± SD).

	Gamma criteria (%) 3 mm/3%	*P* value	gamma criteria (%) 2 mm/2%	*P* value
CBCT	83.7 ± 3.5	0.003	62.2 ± 3.9	0.003
CBCT_cor	96.3 ± 0.8	0.003	91.1 ± 2.4	0.003
SCT1	97.7 ± 0.7	0.004	95.7 ± 1.9	0.021
SCT2	98.7 ± 0.3	–	97.1 ± 0.5	–

## Discussion

In this study, we developed a hybrid approach using conventional phantom correction method and deep learning methods to generate sCT images from CBCT images acquired for NPC patients during their treatments. The HU values of CBCT images were firstly corrected by the HU-ED curves and then used to train the CycleGAN model. For comparison, the original CBCT images were also used for model training with the same parameter settings. Image quality and dose distribution were evaluated on the CBCT images corrected by HU-ED curves and sCT images generated by CycleGAN model, with RCT images as the ground truth.sCT images generated by the CycleGAN model successfully removed most scatter artifacts on the CBCT images. The image quality of SCT1 and SCT2 was visually comparable to the RCT, as Zhang et al.^29^ and Chen et al.^30^ reported. Moreover, the HU profile of sCT images in most regions, especially SCT2, was closer to that of RCT images. It indicated that the HU value of SCT1 and SCT2 images were adequately corrected to that of RCT images, consistent with previous studies^19,31^. The MAE was significantly less in SCT2 than SCT1 (83.51 vs. 105.62, *P* < 0.05), indicating improvement was achieved by training with HU corrected CBCT images. The MAE was greater than previously reported because it was calculated over the structures inside the patient external contour, which was believed to be a better evaluation than calculated from the entire image in previous studies.

The eventual goal of this study is to improve the image quality and dosimetric fidelity for the readily available CBCT obtained during treatment to implement ART for NPC patients if their anatomy changes. Visual inspections of the 3D dose distributions on the sCT images proved it much closer to those calculated from the RCT images, compared to using the uncorrected CBCT and the HU correction only CBCT_cor images. SCT2 was superior to SCT1. As shown in Fig. 4, the prescription dose level isodose line (7350 cGy) from the SCT2 was more close to that of RCT, while the high dose area was obviously larger in the SCT1 images. The average relative dosimetric differences of the SCT2 was significantly lower than that of SCT1 (0.6 vs. 1.2). When evaluated with the 3D gamma analysis, the dose distribution of the SCT2 was more robust than that of SCT1 with a smaller standard deviation (0.5 vs. 1.9) in 2%/2 mm gamma index evaluations.

Our results show that the sCT images generated from the HU corrected CBCT images is superior to that generated from uncorrected CBCT images with the same CycleGAN model, both in image quality and dose calculation accuracy. The sCT images generated by the hybrid method of deep learning and phantom correction could achieve adequate accuracy in ART dose calculations for NPC patients. There were some limitations in this study. First, due to the computational power limitation in hardwares, the current approach was only able to implement the 2D CycleGAN. Even results can be reasonably expected if a three-dimensional model could be adopted^32–34^. Secondly, due to the rigid registration algorithm used for pre-processing, there is a certain difference between RCT images and CBCT images. Deformable image registration may improve the performance of our approach upon validations in future research^35^.

## Conclusion

The image quality and dose calculation accuracy on synthetic CT generated from the deep learning CycleGAN with HU corrected CBCT images were examined and evaluated. This method efficiently provided a 3D volumetric imaging dataset with improved quality and adequate dose calculation accuracy for the application in ART for NPC patients.

## Data Availability

The datasets used and/or analysed during the current study available from the corresponding author on reasonable request.

## References

[1] Chen YP (2021). Chemotherapy in combination with radiotherapy for definitive-intent treatment of stage II-IVA nasopharyngeal carcinoma: CSCO and ASCO guideline. J. Clin. Oncol..

[2] Maheshwari G, Dhanawat A, Kumar HS, Sharma N, Jakhar SL (2020). Clinical and dosimetric impact of adaptive intensity-modulated radiotherapy in locally advanced head-and-neck cancer. J. Cancer Res. Ther..

[3] Ou D (2016). Induction chemotherapy with docetaxel, cisplatin and fluorouracil followed by concurrent chemoradiotherapy or chemoradiotherapy alone in locally advanced non-endemic nasopharyngeal carcinoma. Oral Oncol..

[4] Surucu, M. *et al.* Adaptive radiotherapy for head and neck cancer: Implications for clinical and dosimetry outcomes. *Technol. Cancer Res. Treat.* 1533034616662165 (2016).

[5] Boda-Heggemann J, Lohr F, Wenz F, Flentje M, Guckenberger M (2011). kV cone-beam CT-based IGRT: A clinical review. Strahlenther. Onkol..

[6] Zachiu C (2017). Non-rigid CT/CBCT to CBCT registration for online external beam radiotherapy guidance. Phys. Med. Biol..

[7] Hatton J, Mccurdy B, Greer PB (2009). Cone beam computerized tomography: The effect of calibration of the Hounsfield unit number to electron density on dose calculation accuracy for adaptive radiation therapy. Phys. Med. Biol..

[8] Veiga C (2014). Toward adaptive radiotherapy for head and neck patients: Feasibility study on using CT-to-CBCT deformable registration for “dose of the day” calculations. Med. Phys..

[9] Zllner C, Rit S, Kurz C, Vilches-Freixas G, Landry G (2017). Supplementary materials to “Decomposing a prior-CT-based cone-beam CT projection correction algorithm into scatter and beam hardening components”. Phys. Imaging Radiat. Oncol..

[10] MacFarlane M (2018). Patient-specific calibration of cone-beam computed tomography data sets for radiotherapy dose calculations and treatment plan assessment. J. Appl. Clin. Med. Phys..

[11] Kidar HS, Azizi H (2018). Assessing the impact of choosing different deformable registration algorithms on cone-beam CT enhancement by histogram matching. Radiat. Oncol..

[12] Lei, Y., Wang, T., Liu, Y., Higgins, K. & Yang, X. MRI-based synthetic CT generation using deep convolutional neural network, in *Proceedings of SPIE - The International Society for Optical Engineering*, 100 (2019).

[13] Massa HA, Johnson JM, McMillan AB (2020). Comparison of deep learning synthesis of synthetic CTs using clinical MRI inputs. Phys. Med. Biol..

[14] Yuan N (2020). Convolutional neural network enhancement of fast-scan low-dose cone-beam CT images for head and neck radiotherapy. Phys. Med. Biol..

[15] Kida S (2018). Cone beam computed tomography image quality improvement using a deep convolutional neural network. Cureus.

[16] Li Y (2019). A preliminary study of using a deep convolution neural network to generate synthesized CT images based on CBCT for adaptive radiotherapy of nasopharyngeal carcinoma. Phys. Med. Biol..

[17] Liang X (2019). Generating synthesized computed tomography (CT) from cone-beam computed tomography (CBCT) using CycleGAN for adaptive radiation therapy. Phys. Med. Biol..

[18] Kida S (2020). Visual enhancement of Cone-beam CT by use of CycleGAN. Med. Phys..

[19] Sun H (2021). Imaging study of pseudo-CT synthesized from cone-beam CT based on 3D CycleGAN in radiotherapy. Front. Oncol..

[20] Spadea MF, Maspero M, Zaffino P, Seco J (2021). Deep learning based synthetic-CT generation in radiotherapy and PET: A review. Med. Phys..

[21] Tien HJ, Yang HC, Shueng PW, Chen JC (2021). Cone-beam CT image quality improvement using Cycle-Deblur consistent adversarial networks (Cycle-Deblur GAN) for chest CT imaging in breast cancer patients. Sci. Rep..

[22] Kurz C (2019). CBCT correction using a cycle-consistent generative adversarial network and unpaired training to enable photon and proton dose calculation. Phys. Med. Biol..

[23] Harms J (2019). Paired cycle-GAN-based image correction for quantitative cone-beam computed tomography. Med. Phys..

[24] Zhao J (2021). MV CBCT-based synthetic CT generation using a deep learning method for rectal cancer adaptive radiotherapy. Front. Oncol..

[25] Pinter C, Lasso A, Wang A, Jaffray D, Fichtinger G (2012). SlicerRT: radiation therapy research toolkit for 3D Slicer. Med. Phys..

[26] He, K., Zhang, X., Ren, S. & Sun, J. Deep residual learning for image recognition. In *2016 IEEE Conference on Computer Vision and Pattern Recognition (CVPR)* 770–778 (IEEE, 2016).

[27] Kingma, D. & Ba, J. Adam: A method for stochastic optimization. In *3rd International Conference for Learning Representations* 1–13. arXiv:1412.6980 (2014).

[28] Abadi, M. *et al.* TensorFlow: Large-scale machine learning on heterogeneous distributed systems. In *OSDI'16: Proceedings of the 12th USENIX conference on Operating Systems Design and Implementation* 265–283 (2016).

[29] Zhang Y (2021). Improving CBCT quality to CT level using deep learning with generative adversarial network. Med. Phys..

[30] Chen X (2022). A more effective CT synthesizer using transformers for cone-beam CT-guided adaptive radiotherapy. Front. Oncol..

[31] Zhang Y (2022). Generating synthesized computed tomography from CBCT using a conditional generative adversarial network for head and neck cancer patients. Technol. Cancer Res. Treat..

[32] Kearney V (2019). Attention-enabled 3D boosted convolutional neural networks for semantic CT segmentation using deep supervision. Phys. Med. Biol..

[33] Li Y (2021). VolumeNet: A lightweight parallel network for super-resolution of MR and CT volumetric data. IEEE Trans. Image Process. Publ. IEEE Signal Process. Soc..

[34] Hsu K (2022). Improving performance of deep learning models using 35D U-Net via majority voting for tooth segmentation on cone beam computed tomography. Sci. Rep..

[35] Cao X, Gao Y, Yang J, Wu G, Shen D (2016). Learning-based multimodal image registration for prostate cancer radiation therapy. Med. Image Comput. Comput. Assist. Interv..

